# Extracellular vesicles from *Cutibacterium acnes* phylotypes IA1, IB, II and III, which contain glucoglycerolipids, are not cytotoxic but induce a strong pro-inflammatory response in human prostate epithelial and fibroblast cells

**DOI:** 10.1186/s12866-026-05490-x

**Published:** 2026-08-06

**Authors:** Anna Chudzik, Marzena Ciesielska, Marta Kaszowska, Katarzyna Kapczyńska, Katarzyna Pacyga-Prus, Wojciech Jachymek, Andrzej Gamian, Mariola Paściak

**Affiliations:** 1 Laboratory of Medical Microbiology, Hirszfeld Institute of Immunology and Experimental Therapy, Polish Academy of Sciences, Rudolfa Weigla 12, Wroclaw, 53- 114 Poland; 2 Laboratory of Microbial Immunochemistry and Vaccines, Hirszfeld Institute of Immunology and Experimental Therapy, Polish Academy of Sciences, Rudolfa Weigla 12, Wroclaw, 53-114 Poland; 3 Laboratory of Microbiome Immunobiology, Hirszfeld Institute of Immunology and Experimental Therapy, Polish Academy of Sciences, Rudolfa Weigla 12, Wroclaw, 53-114 Poland

**Keywords:** Glycolipids, Glycerolipids, *Cutibacterium acnes*, *C. acnes* phylotypes, Extracellular vesicles, Prostate cells

## Abstract

*Cutibacterium acnes* represents one of the predominant bacterial taxa colonizing human skin. Its impact on host physiology is strongly phylotype-dependent: certain phylotypes are associated with the initiation and progression of acnes lesions, whereas others contribute to the stability and homeostasis of the cutaneous microbiota. Moreover, phylotype II *C. acnes* has been increasingly implicated in the etiology of chronic prostatitis, suggesting a broader pathogenic potential. The growing incidence of antimicrobial resistance in *C. acnes* underscores the need to identify novel antigenic determinants and molecular candidates for next-generation vaccine strategies. Notably, *C. acnes*, like many Gram-positive bacteria, produces extracellular vesicles (EVs) that function as carriers of enzymes, metabolites, immunogenic components and lipid structures.

In the present study, four glycolipids (GL1–GL4) isolated from distinct *C. acnes* phylotypes were subjected to comprehensive structural characterization using GLC-MS, MALDI-TOF MS and NMR spectroscopy. These GL1-GL4 compounds are glycoglycerolipids composed of one or two glucose residues, a glycerol moiety and different numbers of fatty acid residues (mainly 15:0–18:0). The presence of GL1 within EVs fractions obtained from four *C. acnes* phylotypes has been confirmed. Subsequently, the effects of EVs and the glycolipids GL1 and GL3 on the viability of human prostate epithelial and fibroblast cell lines were assessed. None of the tested preparations exhibited significant cytotoxicity. However, EVs derived from phylotypes IA1 and II elicited markedly elevated release of IL-6 and TNF-α, demonstrating their substantial pro-inflammatory capacity and supporting their potential role in host-pathogen interactions.

**Supplementary Information:**

The online version contains supplementary material available at https://doi.org/10.1186/s12866-026-05490-x.

## Highlights

*Cutibacterium acnes* is part of the skin microbiota, whose biological activity is strongly phylotype-dependent and is increasingly associated with chronic prostatitis. The rising prevalence of antimicrobial resistance necessitates the identification of novel antigenic and molecular targets. In this study, four glycolipids (GL1–GL4) were isolated and structurally characterized across distinct *C. acnes* phylotypes, significantly enhancing our understanding of its lipid architecture. These glycoglycerolipids comprise glucose residues, a glycerol backbone, and predominantly 15:0–18:0 fatty acid residues. Importantly, GL1 was detected within extracellular vesicles (EVs) derived from all analyzed phylotypes, indicating selective incorporation into vesicular cargo. Our investigations demonstrated that EVs from phylotypes IA1 and II markedly increased IL-6 and TNF-α secretion in human prostate epithelial and fibroblast cell lines, confirming pronounced pro-inflammatory capacity. Notably, this study provides the first evidence that *C. acnes* type II modulates prostate cell responses through EV-mediated mechanisms, revealing a previously uncharacterized pathway of host-pathogen interaction.

**Supplementary Information:**

The online version contains supplementary material available at https://doi.org/10.1186/s12866-026-05490-x.

## Introduction

*Cutibacterium acnes* are Gram-positive, facultative anaerobic microorganisms that are part of the human skin microbiota. This species, along with *Staphylococcus epidermidis*, is the most abundant microorganism in humans due to its unique adaptations that allow it to thrive on skin rich in sebaceous glands [1]. These bacteria, especially phylotypes IB and III *C. acnes*, are a natural component of healthy skin microbiota, preventing the excessive expansion of pathogens, which is why their presence on the skin is crucial [1]. Although *C. acnes* is usually associated with common skin disease, *acne vulgaris*, the pro-inflammatory potential is closely related to individual phylotypes belonging to this species. Eight phylotypes have already been identified within *C. acnes*, and their varying degrees of association have been demonstrated not only with the occurrence of acne lesions but also with other diseases, including prostatitis [2, 3]. So far, the *C. acnes* phylotype most closely associated with acne lesions was IA1, while phylotype II is most frequently identified in samples collected from prostate tissues. Other *C. acnes* phylotypes, such as IB and III, are correlated with healthy skin [4]. The differentiation between acne-associated *Cutibacterium acnes* strains (predominantly phylotype IA1) and healthy skin-associated strains (phylotypes IB, II, and III) relies on distinct genetic, metabolic, and phenotypic virulence profiles: hyper-inflammatory phylotype IA1 strains exhibit a significantly greater capacity to induce pro-inflammatory cytokines (such as IL-6, TNF-α, and IL-8) and form robust biofilms that contribute to follicular plugging [5]. *C. acnes* isolates from both skin and implants belonging to IA1 phylotype, produced significantly more biofilm than other phylotypes, and this capacity was independent of the anatomical site of isolation [6]. Subsequent studies of *C. acnes* biofilms confirmed that IA1 strains are stronger biofilm formers and generate more structurally complex biofilms than phylotype II strains [7].

Furthermore, the acne-associated strains produce much higher levels of porphyrins and lipases, which degrade sebum into irritating free fatty acids, compared to the more benign phylotypes II and III [8].

A comprehensive comparative analysis of 255 *C. acnes* genomes was carried out to identify unique features defining the different phylotypes [9]. Pangenome analysis revealed distinct sets of unique genes for types I, II, and III. Type I strains possess unique carbohydrate metabolism and transporter genes, distinct hyaluronate lyase (HYL) alleles, and lack the porphyrin-biosynthesis repressor in the highly virulent IA1 sub-clade [9, 10]. Conversely, type III strains lack HYL, harbor more genes overall [9], and are associated with noninflammatory skin conditions [11, 12]. Furthermore, CRISPR-Cas systems were detected exclusively in type II strains and were absent from both type I and type III [9].

A recent genome-wide association study on infection-associated and commensal isolates of *C. acnes* revealed that all phylotypes of *C. acnes*, except phylotype III, can cause deep-seated infection under favorable conditions, and that genetic content appears to have only a minor effect on the likelihood of infection establishment [13].

Ultimately, it is the loss of phylotype diversity and the clonal expansion of these hyper-virulent IA1 lineages rather than the total bacterial abundance that drives acne pathogenesis. It is essential to study different *C. acnes* phylotypes individually, as was demonstrated that they vary in crucial structural components such as proteins and lipids [14, 15].

Lipids are the basic building blocks of cell membranes, and at the same time, they can constitute a cellular fingerprint that allows for the differentiation of bacteria even at the level of phylotypes belonging to the same species [16]. However, bacterial lipids may also be considered antigenic compounds, depending on their structural characteristics. Lipid antigens include primarily polar lipids containing headgroups that can be presented on the CD1 surface and specifically recognized by the T cell receptor (TCR) [17]. This applies to glycolipids containing sugar residues in their structure, such as diacylglycerol glycolipids, which are bound by CD1d and presented for recognition by NKT cells [18, 19].

Extracellular vesicles (EVs) are spherical nanoparticles produced by both prokaryotic and eukaryotic cells. The mechanisms of bacterial EVs biogenesis differ between Gram-positive and Gram-negative bacteria. In Gram-negative bacteria, EVs are formed primarily by budding of the outer membrane. Gram-positive bacteria produce EVs by bulging the cell membrane and local peptidoglycan digestion, allowing their release into the extracellular environment [20]. EVs are composed of a phospholipid bilayer and may contain a whole range of bacterial antigens, including proteins, toxins, and cellular metabolites. The composition of EVs secreted by *C. acnes* is unique for individual phylotypes, both in terms of protein and lipid profiles, contributing to distinct effects on eukaryotic cells [14]. EVs from *C. acnes* IA1, exhibit a unique, protein-rich cargo including survival-related chaperones (GroEL, DnaK) and surface proteins—that functions as PAMPs to drive severe inflammation. This molecular payload induces a potent pro-inflammatory response in human skin cells, triggering the increased expression of cytokines IL-6, IL-8, TNF-α, and GM-CSF, comparing to IB phylotypes [2, 21]. Although detailed characterization of EVs from phylotype II and III remains limited, currently available evidence suggests that EV composition and host immunomodulatory activity are phylotype-dependent [15].

The thick layer of peptidoglycan that builds the cell walls of *C. acnes* prevents the exposure of glycolipids located in the cell membrane to the host’s immune system. Due to the mechanism of EVs biogenesis in Gram-positive microorganisms, these nanostructures have a lipid bilayer composition specific to the cell membranes from which they originate. These features make the presentation of bacterial membrane glycolipids possible through EVs. The objective of this study was to conduct a structural analysis of glycolipids isolated from four phylotypes of *C. acnes* (IA1, IB, II, and III). The interactions of glycolipids with human fibroblast and prostate epithelial cells in vitro were then evaluated. Furthermore, the objective was to identify these glycolipids in extracellular vesicles released by *C. acnes* and to analyze the interactions of these vesicles with the selected human cell lines.

The gold standard for the structural analysis of lipid molecules currently involves techniques based on mass spectrometry (MS) and nuclear magnetic resonance (NMR) spectroscopy. Fatty acids and the sugar residues that form glycolipids are effectively derivatized into volatile derivatives, which are then analyzed using gas-liquid chromatography-mass spectrometry (GLC-MS). When combined with the fragmentation obtained from MALDI-TOF MS spectra and supplemented by NMR analysis, this approach provides a comprehensive understanding of the molecule’s structure.

Although bacterial protein antigens have been extensively studied in vaccine development, relatively little attention has been given to lipids, mainly due to their structural properties. This work not only identifies the optimal techniques for structurally characterizing bacterial glycolipids isolated from *C. acnes* but also assesses their potential to induce an immune response, particularly the expression of pro-inflammatory cytokines, using both purified glycolipids and EVs naturally produced by these bacteria.

## Materials and methods

### Bacterial strains

The present study utilized four *C. acnes* phylotypes: IA1 (DSM 1897), IB (DSM 16379), II (PCM 2334) and III (NCTC 13655). The strains were obtained from DSM - German Collection of Microorganisms and Cell Cultures, PCM - Polish Collection of Microorganisms, and NCTC - National Collection of Type Cultures (United Kingdom).

### Bacterial growth conditions and EVs isolation

All *C. acnes* phylotypes were cultivated as previously described [14, 16]. Briefly, after cultivating under anaerobic conditions at 37 °C in thioglycollate-soy broth mixed with trypticasein soy broth (1:1, v/v) and reaching the mid-exponential growth phase, bacteria were harvested by centrifugation (4,000 x g, 4 °C, 10 min) and cells were washed thrice with PBS and Milli-Q water. The supernatants were used to isolate EVs according to the protocol described by Chudzik et al. [14]. To obtain EVs, the supernatant was filtered using a bottle-top 0.22 μm filter, and ultrafiltration of the supernatant was carried out using an Amicon^®^ ultrafiltration system with 100 kDa Ultrafiltration Discs. Then, the concentrate was centrifuged at 4,000 × g for 15 min at 4 °C, and the obtained supernatant was collected and subjected to another centrifugation (15,000 × g, 15 min, 4 °C) followed by ultracentrifugation (100,000 × g, 2 h, 4 °C). The pellet of bacterial EVs was suspended in 1-1.5 ml of PBS (phosphate-buffered saline) and subjected to density gradient purification using 10 to 45% OptiPrep^®^(Iodixanol; Serumwerk, Germany) in PBS, with the EVs sample loaded on top and ultracentrifuged (140,000x g, 16 h, 4 °C). Then, 1.5 mL fractions were collected and dialysed into Milli-Q water using a Pur-A-Lyzer dialysis kit (Sigma-Aldrich, St. Louis, USA) for 72 h, and ultracentrifuged (181,000 × g, 2 h, 4 °C). Individual fractions of 200 µL were collected. The presence of EVs in each fraction, as well as their size, was analyzed using DLS (Malvern Zetasizer, Malvern, UK). The concentration of the EVs was determined using the bicinchoninic acid (BCA) protein assay (Pierce™ BCA Protein Assay Kit, Thermo Fisher, Rockford, IL, USA).

*C. acnes* EVs preparations were extracted according to the Bligh-Dyer method [22].

### EVs standardization

All EV preparations were obtained from the same culture conditions medium to obtain high repeatability. EVs were standardized based on their total protein concentration, which was determined using the BCA (Bicinchoninic Acid) assay. Equal amounts of EV protein were applied to ensure precise and uniform dosing. Additionally, before quantification and functional assays, the size distribution and quality (purity and homogeneity) of the isolated EVs were rigorously verified using Dynamic Light Scattering (DLS) analysis. This confirmed that the preparation consisted of intact vesicles within the expected nanometer range, without significant protein aggregation.

### Crude lipid preparation and analysis

Wet cell mass (100 g) samples of the four *C. acnes* phylotypes were treated twice with chloroform/methanol (2:1, v/v, 1500 ml) at 37 °C for 12 h. The presence of glycolipids in crude lipid extracts was assessed using thin-layer chromatography (TLC) and visualized with orcinol reagent followed by heating at 120 °C according to published protocols [23].

### Glycolipid purification

*C. acnes* lipid extracts were purified using the method described by Paściak et al. [23]. Crude lipids were loaded on a column (1.8 × 60 cm) of activated (at 120 °C) Silica Gel 60 (230–400 mesh, Merck, Darmstadt, Germany) and eluted successively with chloroform, acetone and methanol (4 × 100 ml each solvent). The eluates were monitored for polar lipids by TLC. The methanol fractions that contained glycolipids were further fractionated on a column (1.5 × 45 cm) of Silica Gel 60 (200–300 mesh) with the step gradient of methanol in chloroform (0, 10, 20, 25, 30, 35, 40, 50, 70, 100 vol %) at a flow rate of 1 ml/min. Fractions of 50 ml were collected, and their purity was verified by TLC. The final purification step was performed using preparative TLC [23].

### Chemical analysis

#### Fatty acid residues (FAME analysis)

Purified glycolipids were subjected to acidic methanolysis (2 M methanolic HCl solution, 1 h, 80 °C) [16]. Then, 1.5 ml of Milli-Q water and 1.5 ml of hexane were added to each sample and extracted. The samples were centrifuged (4,000 x g, 10 min, RT) to separate the phases. The upper hexane phase was collected in a separate tube, and the aqueous phase was re-extracted by adding 1 ml of hexane. The hexane phases were combined and evaporated under a stream of nitrogen. Before GLC-MS analysis, the obtained FAMEs (fatty acid methyl esters) were dissolved in 100 µl of hexane. The injection volume was 1 µl. Each sample was measured three times. Analysis was performed using a Focus GC with a Zebron ZB-5HT Phenomenex column (30 m × 0.25 mm × 0.25 μm w/5 m Guardian) combined with an ITQ 700 (Thermo Scientific ITQ Series) mass detector. The carrier gas was helium with a 0.3 ml/min flow rate. The mass range was 50–500 *m/z*, with positive polarity. Separation gradients in gas chromatography were set as follows: starting at 150 °C for 4 min and then increasing by 12 °C/min to reach 270 °C. The raw mass spectrometry data were processed using Thermo Xcalibur software [16]. FAMEs were identified by their peak retention time and mass spectra, and were compared against a reference standard FAMEs test mix (SUPELCO 37-Component FAME Mix, Bellefonte, PA).

#### Carbohydrate residues

To obtain information about sugar residues present in *C. acnes* glycolipids, the acid hydrolysis and derivatisation of the monosaccharides to alditol acetates were performed [24]. The glycolipid samples were hydrolyzed using 1 M HCl at 100 °C for 5 h. The acid was then removed by washing twice with 300 µL Milli-Q water and once with 300 µl methanol. After each rinse, the contents of the tubes were evaporated under a stream of nitrogen. Next, 1 ml of chloroform and 1 ml of Milli-Q water were added to the samples, vortexed and then centrifuged (4,000 x g, 5 min) to separate the phases. The water phase was transferred to a new test tube with 30 mg of sodium borohydride and subjected to overnight reduction at 4 °C. Excess borohydride was removed by dropwise addition of acetic acid and then evaporated under a stream of nitrogen. The samples were washed several times with methanol and incubated at 100 °C for 35 min with the addition of pyridine and acetic anhydride (200 µl each). In the next step, the pyridine was evaporated, and the samples were washed several times with toluene. In the last step, Milli-Q water and chloroform were added to the samples and the lower phase was extracted several times, collecting the polar phase in a separate tube. Before analysis, each sample was suspended in 100 µl of ethyl acetate. The alditol acetates were detected by GLC-MS (Thermo Scientific ITQ 700 Focus GC) by applying the same temperature gradient as for fatty acid analysis. Thermo Xcalibur software was used to analyze the data. Identification of alditol acetates was made based on peak retention times and by comparing the obtained mass spectra with authentic monosaccharide standards.

### NMR analysis

NMR spectra were recorded using a Bruker Avance III 600 MHz spectrometer using a 5 mm QCI ^1^H/^13^C/^15^N/^31^P probe equipped with a *z*-gradient. The measurements were performed at 298 K. Purified glycolipids (GL1-GL4) were dissolved in CDCl_3_/CD_3_OD (2:1, v/v). The acetone (δ_H_/δ_C_ 2.225/31.05 ppm) was used as an internal reference. The data were acquired and processed using Bruker Topspin software 3.1 and with the help of the SPARKY program *(Goddard & Kneller*,* 2001).* The signals were assigned by one-dimensional (1D) and two-dimensional (2D) experiments: correlation spectroscopy (COSY), total correlation spectroscopy (TOCSY, with mixing time: 30, 60 and 100 ms), Heteronuclear Single Quantum Coherence – Distortionless Enhancement by Polarization Transfer (HSQC-DEPT) and nuclear Overhauser effect spectroscopy (NOESY, with mixing time of 100 ms).

### MALDI-TOF mass spectrometry

Intact glycolipids were analyzed by MALDI-TOF (Matrix-Assisted Laser Desorption/Ionization Time-of-Flight) mass spectrometry using an ultrafleXtreme III instrument (Bruker Daltonics, Bremen, Germany). All glycolipid samples were dissolved in chloroform-methanol (2:1, v/v) at a concentration of 1 mg/ml and then applied onto a target plate (MTP 384 ground steel BC, Bruker, Billerica, MA, USA). Norharmane (Sigma-Aldrich) in chloroform-methanol (2:1, v/v) at a concentration of 5 mg/ml was used as a matrix. The analysis was performed in the reflectron positive ion mode. Peptide Calibration Standard II (Bruker Daltonics, Bremen, Germany) was used for calibration purposes. Fragmentation spectra were obtained using the ultrafleXtreme instrument with LIFT technology, and all collected data were analyzed with Bruker Daltonics Flex Analysis Software (version 3.4).

### ESI-IT mass spectrometry

The ESI mass spectra were acquired on ESI-IT (Electrospray Ionization - Ion Trap) amaZon SL (Bruker Daltonik GmbH, Bremen, Germany) in negative-ion mode with 100–2200 *m/z* scan range. External calibration of the mass spectrometer was performed using ESI Tuning Mix (Agilent Technologies, Santa Clara, CA, USA) in negative-ion mode before analysis. Glycolipids were dissolved in chloroform: methanol (2:1 (v/v), 100 µg/ml) and directly injected into the ESI source at a flow rate of 1 µl/min using a syringe pump. The source parameters were as follows: source temperature, 200 °C; nitrogen flow, 5 l/min at 8 psi.

Fragmentation analysis (MS/MS) was carried out on ESI-IT amaZon SL (Bruker Daltonik GmbH, Bremen, Germany) in negative ion mode. MS^n^ experiments were acquired in the 100–2000 *m/z* range using an isolation window of 4 *m/z*, an amplitude value of 0.35, and SmartFrag mode of 60–120%.

### Human cell lines and cell culture

The human fibroblast cell line MSU-1.1 (CVCL_9S81) was kindly gifted by C. Grillon from the Centre National de la Recherche Scientifique, France [25]. The prostate epithelial PWR-1E (CRL-3661) cell line was purchased from the American Type Culture Collection (ATCC) [26].

Human fibroblast cell line MSU-1.1 was grown in DMEM medium supplemented with 10% Fetal Bovine Serum (FBS), 1% penicillin/streptomycin solution (Sigma-Aldrich) and 2mM L-Glutamine (Sigma-Aldrich). Human epithelial prostate cell line PWR-1E was grown in Keratinocyte Serum-Free Medium with L-Glutamine, EGF (epidermal growth factor), and BPE (bovine pituitary extract) supplied separately (Gibco). The cells were cultured at 37 °C in an atmosphere of 95% relative humidity and 5% CO_2_. Cells were passaged using Accutase^®^ solution (Sigma-Aldrich).

### Cell viability

Cell proliferation was investigated using the standard 3-(4,5-dimethylthiazol-2-yl)-2,5-diphenyltetrazolium bromide (MTT, Sigma-Aldrich) assay protocol for cell viability. Briefly, 1 × 10^4^ human cells were seeded onto separate 96-well plates in triplicate. For the MSU-1.1 cell line, after adhering for 24 h, the medium was changed to serum-reduced (5%) DMEM, while the PWR-1E line remained in the culture medium. In the next step, EVs obtained from *C. acnes* (IA1, IB, II, and III phylotypes) were added to each cell line at final concentrations ranging from 6.25 µg/ml to 100 µg/ml. In parallel, the effect of two main *C. acnes* glycolipids (GL1 and GL3) solutions in PBS with Tween 20 (0.00004–0.04%) was also studied in final concentrations ranging from 0.0002 µg/ml to 20 µg/ml. All concentrations of both EVs and glycolipids were applied to human cells in triplicate in 2 independent experiments. Cells not treated with either vesicles or glycolipids were used as a control. After stimulation for 24 h, 50 µl of supernatants from each well were collected, centrifuged to remove any cells, and frozen at -20 °C for further analysis.

### Proinflammatory cytokine levels

The secretion of proinflammatory cytokines: IL-1β, IL-6 and TNF-α was determined using the Milliplex Human Cytokine Panel (Millipore, Merck, Darmstadt, Germany) on the Luminex 200™ System (Merck, Darmstadt, Germany) according to the manufacturer’s protocols.

### Statistical analysis

Statistical analyses were performed with GraphPad Prism 5.01 software. The values for all measurements are presented as the mean ± standard deviation (SD). The viability of cell lines stimulated with GLs and EVs was evaluated using a two-way ANOVA followed by Dunnett’s multiple comparisons test. Proinflammatory cytokine levels relative to the non-stimulated control were analyzed using a two-way repeated measures (RM) ANOVA, followed by a Bonferroni post hoc test (ns: *p* > 0.05, *p < 0.05, **p < 0.01, ***p < 0.001 vs. non-stimulated controls). Additionally, comparisons of cytokine levels among the phylotypes themselves were performed using a two-way ANOVA followed by Tukey’s post hoc test (*p < 0.05, **p < 0.01, ***p < 0.001, ****p < 0.0001). Pairs without a label/asterisks were non-significant (*p* > 0.05).

## Results

### Chemical analysis of glycolipids

**Fig. 1:**
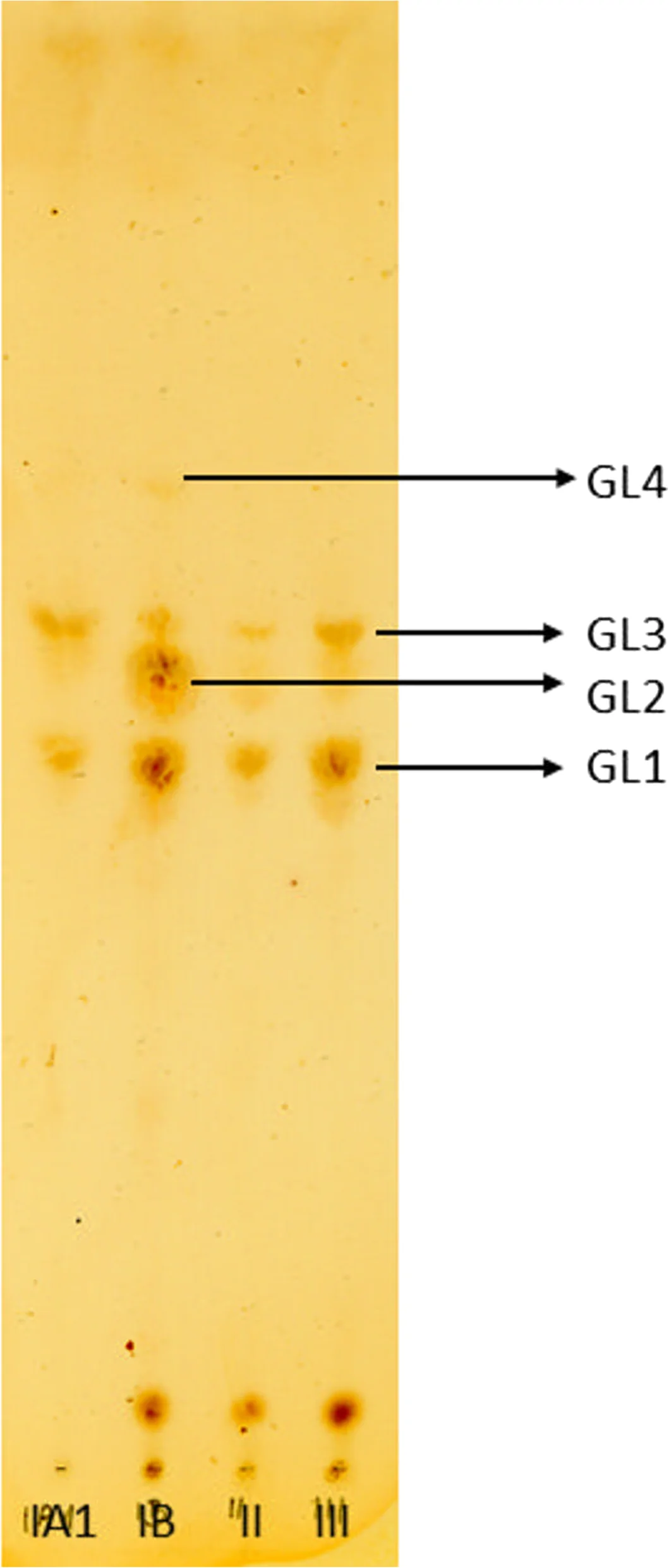
TLC glycolipid profiles of *Cutibacterium acnes* IA1 – DSM 1897, IB – DSM 16,379, II – PCM 2334 and III – NCTC 13,655. The TLC was developed in the solvent system: chloroform-methanol-water (65:25:4, v/v/v), and for the detection of glycolipids, orcinol reagent was applied

As a result of chloroform-methanol extraction of the *C. acnes* DSM 1897, DSM 16,379, PCM 2334 and NCTC 13,655 cells, approximately 400–500 mg of each crude lipid extract was obtained. The presence of glycolipids in the crude lipid extract was confirmed by TLC, visualised with vanillin and orcinol reagents. It has been shown that two major glycolipids (GL1 and GL3) are present in all tested *C. acnes* phylotypes (Fig. 1). In the case of phylotype IB (*C. acnes* DSM 16379), the presence of four glycolipids (GL1-GL4) was demonstrated. Their numbering depended on the retention factor (RF) for individual glycolipids: GL1, 0.47; GL2, 0.52; GL3, 0.56; GL4, 0.65 (Fig. 1). As a result of the purification of crude lipid extracts using column chromatography, it was possible to separate glycolipids from phospholipids and nonpolar lipids. Also, the purification procedure made glycolipid GL4 more visible.

GLC-MS analysis of FAMEs revealed that all glycolipids contain saturated fatty acid residues *iso* and *anteiso*-methyl branched, similar to those characteristic of *C. acnes* cells [16], which include tetradecanoic (14:0), pentadecanoic (15:0), hexadecanoic (16:0), heptadecanoic (17:0), and octadecanoic (18:0) (Table 1). The analysis of the alditol acetates showed that GL1-GL4 glycolipids contained only glucose residues as a carbohydrate component.

**Table 1:** Fatty acid content in *C. acnes* glycolipids (GL1-GL4) based on GLC-MS analysis

Fatty acid	GL1	GL2	GL3	GL4
	%			
*i *14:0	-	-	0.47	-
*i* 15:0	46.66	36.96	60.20	31.52
*a.i*. 15:0	7.42	3.62	8.52	6.31
*n* 15:0	2.59	3.83	3.64	1.72
*a.i*. 16:0	0.37	-	0.55	-
*n* 16:0	11.80	17.98	4.48	25.68
*i *17:0	15.00	10.17	16.35	4.59
*a.i*. 17:0	3.08	-	3.21	0.99
*n* 17:0	1.04	1.21	0.78	-
*n* 18:0	12.04	26.24	1.81	29.19

*Abbreviation: i. iso* methyl branched, *a.i. anteiso* methyl branched, *n* normal, *i.e.* straight chain fatty acids

**Fig. 2:**
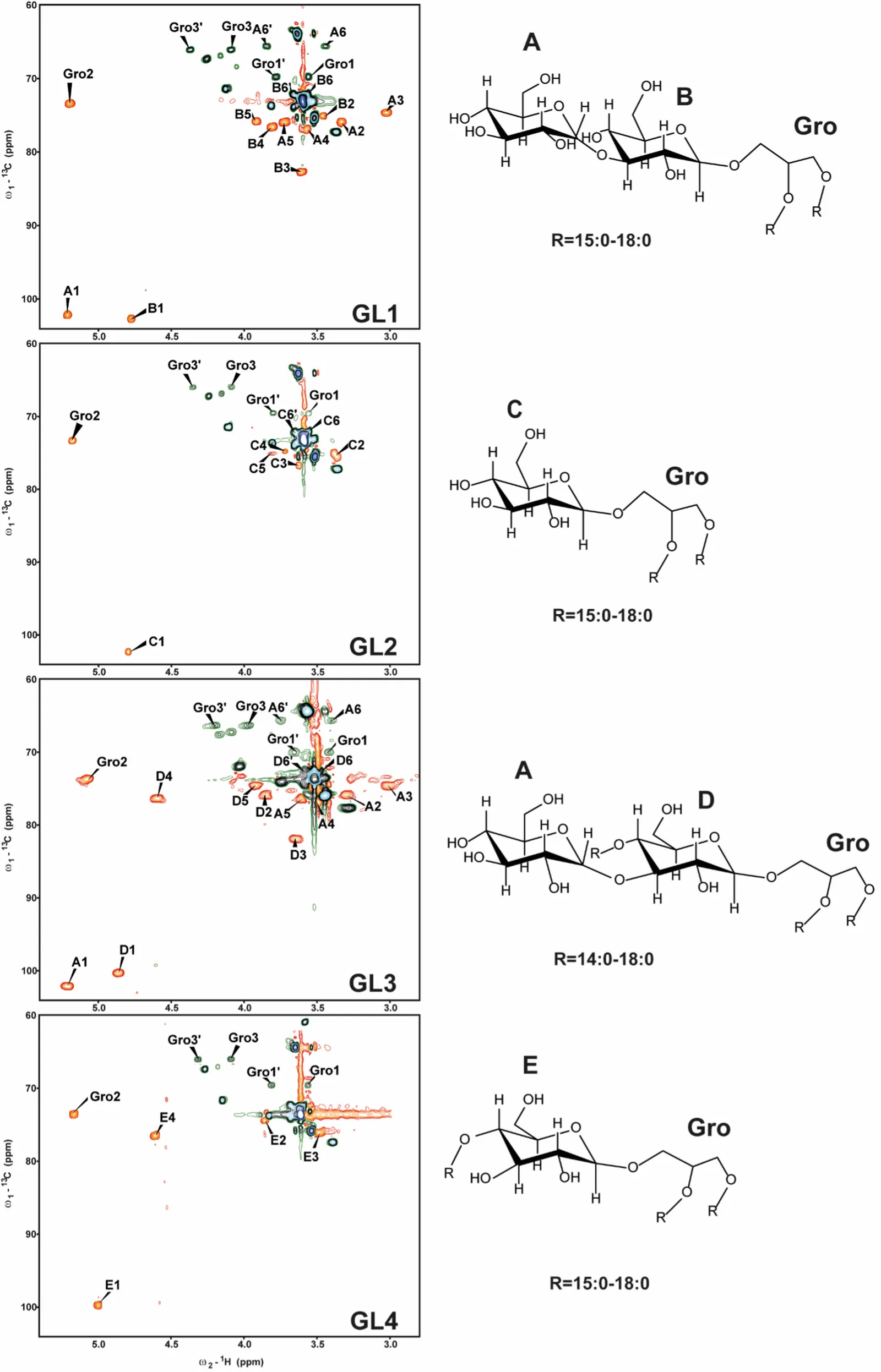
Selected ^1^J_H, C_- and ^3^J_H, C_ -connectivities in HSQC-DEPT spectra of the *C. acnes* glycolipids (GL1-GL4)

The initial NMR investigation of the *C. acnes* glycolipids indicated the presence of glucose (Glc), glycerol (Gro) and fatty acid residues. The GL1-GL4 were analyzed by ^1^H, ^13^C and ^31^P NMR spectroscopy. The ^31^P NMR spectra did not indicate the presence of phosphate groups in GL1-GL4. The ^1^H NMR spectra of the GL1-GL4 contained main signals for two anomeric protons (GL1 and GL3), and one anomeric proton (GL2 and GL4), as well as signals characteristic of the glycerol and fatty acid residues. The ^1^H-^1^H COSY, ^1^H-^1^H TOCSY (using different mixing times), allowed for the assignment of the H1 to H6,6’ signals for each sugar and glycerol residues, whereas ^1^H-^13^C HSQC-DEPT spectra contained signals also for carbons (Table 2; Fig. 2).

**Table 2:** ^1^H and ^13^C NMR chemical shifts of *C. acnes* glycolipids (GL1-GL4)

				Chemical shifts (ppm)					
				H1, (H1’) C1	H2 C2	H3, (H3’) C3	H4 C4	H5 C5	H6, (H6’) C6
GL1	A	α-D-Glc*p*	5.21 102.1		3.32 75.8	3.02 74.8	3.57 76.7	3.73 76.1	3.43, 3.85 65.6
	B	→3)-β-D-Glc*p*	4.78 102.6		3.46 75.1	3.60 82.7	3.81 76.8	3.92 75.8	3.57, 3.66 72.0
		1)-Gro	3.56, 3.78 69.9		5.20 73.4	4.10, 4.37 66.2			
GL2	C	β-D-Glc*p*	4.82 102.6		3.38 75.6	3.41 75.4	3.65 76.9	3.97 71.8	3.56, 3.65 72.3
		1)-Gro	3.63, 3.83 69.8		5.20 73.7	4.10, 4.36 66.2			
GL3	A	α-D-Glc*p*	5.21 102.3		3.29 76.1	3.62 76.4	3.47 77.7	3.59 76.6	3.40, 3.74 65.8
	D	→3,4)-β-D-Glc*p*	4.87 100.3		3.86 76.1	3.64 82.1	4.60 76.6	3.92 74.5	3.60, 3.68 72.3
		1)-Gro	3.42, 3.65 69.9		5.10 73.5	3.97, 4.21 66.0			
GL4	E	→4)-β-D-Glc*p*	5.00 99.8		3.84 74.4	3.47 76.2	4.60 76.5	nd	nd
		1)-Gro	3.56, 3.81 69.8		5.16 73.5	4.08, 4.30 66.2			

*nd* not detected

GL1. Residue A with H1/C1 signals at δ_H_/δ_C_ 5.21/102.1 ppm (^1^*J*_C−1,H−1_ = 172 Hz), was assigned as the terminal α-D-Glc*p* residue based on the large couplings among all protons in the spin system. Residue B with H1/C1 signals at δ_H_/δ_C_ 4.78/102.6 ppm (^1^*J*_C−1,H−1_ = 163 Hz), was recognized as 3-substituted β-D-Glc*p*. A significant downfield shift was observed for C3 (δ_C_ 82.7 ppm) as compared with the chemical shift of the corresponding non-substituted monosaccharide. Residue Gro with H1,1’/C1 signals at δ_H, H’_/δ_C_ 3.56, 3.78/69.9 ppm, H2/C2 signals at δ_H_/δ_C_ 5.20/73.4 ppm, and H3,3’/C3 signals at δ_H, H’_/δ_C_ 4.10, 4.37/66.2 ppm, was identified as glycerol residue (Gro). The chemical shifts were inferred by comparison with published data [27]. Thus, the carbohydrate moiety of the GL1 has the following structure: α-D-Glc*p*-(1→3)-β-D-Glc*p*-(1→1)-Gro diester, where O-acyl esters attached to the glycerol moiety include fatty acids (from 15:0 to 18:0 as was identified above).

GL2. Residue C with H1/C1 signals at δ_H_/δ_C_ 4.82/102.6 ppm (^1^*J*_C−1,H−1_ = 162 Hz), was recognized as terminal β-d-Glc*p* based on the large vicinal couplings between all protons in the sugar ring [28]. Residue Gro was assigned as glycerol with H1,1’/C1 signals at δ_H, H’_/δ_C_ 3.63, 3.83/69.8 ppm, H2/C2 signals at δ_H_/δ_C_ 5.20/73.7 ppm, and H3,3’/C3 signals at δ_H, H’_/δ_C_ 4.10, 4.36/66.2 ppm [27]. GL2 has the following structure: β-D-Glc*p*-(1→1)-Gro diester, where O-acyl esters attached to the glycerol moiety include fatty acids (from 15:0 to 18:0 as was identified above).

GL3. Residue A with H1/C1 signals at δ_H_/δ_C_ 5.21/102.3 ppm (^1^*J*_C−1,H−1_ = 172 Hz), was recognized as terminal α-D-Glc*p* based on the large couplings among all protons in the sugar ring (similarly as in GL1). Residue D with H1/C1 signals at δ_H_/δ_C_ 4.87/100.3 ppm (^1^*J*_C−1,H−1_ = 162 Hz) have been identified as a 3,4-disubstituted β-D-Glc*p* residue based on the characteristic chemical shift of the C3 (δ_C_ 82.1 ppm) and H4 (δ_H_ 4.60 ppm) as compared with the chemical shift of the corresponding non-substituted monosaccharide. The substitution by fatty acid residue has been identified at C4. Residue Gro was assigned as glycerol with H1,1’/C1 signals at δ_H, H’_/δ_C_ 3.42, 3.65/69.9 ppm, H2/C2 signals at δ_H_/δ_C_ 5.10/73.5 ppm, and H3,3’/C3 signals at δ_H, H’_/δ_C_ 3.97, 4.21/66.0 ppm. GL3 has the structure: α-D-Glc*p*-(1→3)-β-D-Glc*p*-(1→1)-Gro triester, where O-acyl esters attached to the β-D-Glc*p* (residue D) at C4 and glycerol moiety include fatty acids (mainly from 14:0 to 18:0, as was identified above).

GL4. Residue E with H1/C1 signals at δ_H_/δ_C_ 5.00/99.8 ppm (^1^*J*_C−1,H−1_ = 162 Hz), was recognized as 4-substituted β-d-Glc*p*. Relative downfield chemical shifts of the C4 at δ_C_ 76.5 ppm indicated the substitution position by fatty acid residue. Residue Gro was assigned as glycerol with H1, 1’/C1 signals at δ_H, H’_/δ_C_ 3.56, 3.81/69.8 ppm, H2/C2 signals at δ_H_/δ_C_ 5.16/73.5 ppm, and H3,3’/C3 signals at δ_H, H’_/δ_C_ 4.08, 4.30/66.2 ppm. The GL4 structure: β-D-Glc*p*-(1→1)-Gro triester, where O-acyl esters attached to the glycerol moiety include fatty acids (mainly from 15:0 to 18:0 as was identified above) and β-D-Glc*p* at the C4 position.

**Fig. 3:**
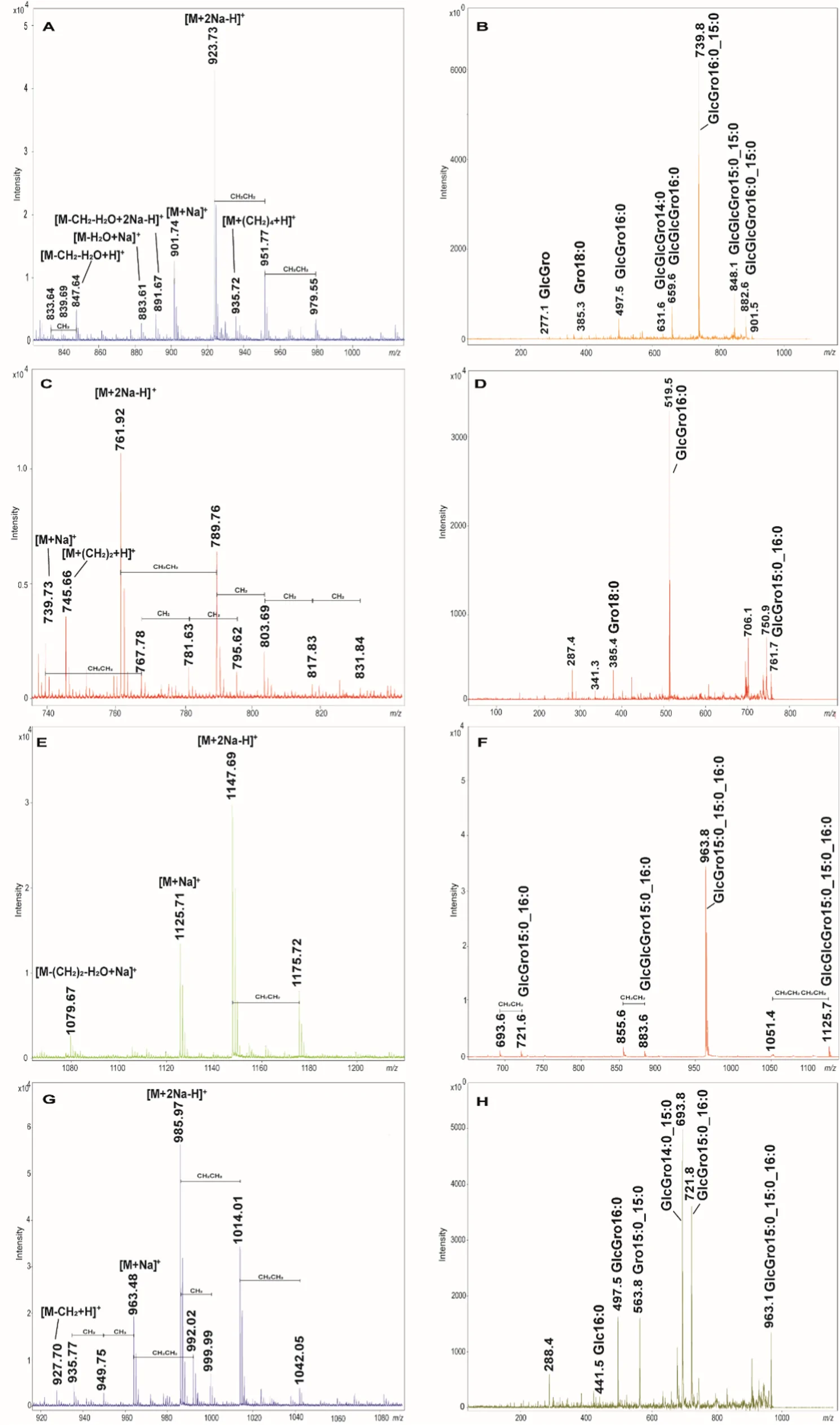
MALDI-TOF MS spectra of the *C. acnes* glycolipids GL1 (**A**), GL2 (**C**), GL3 (**E**), GL4 (**G**), and MALDI-TOF MS/MS spectra of ions selected for further analysis: *m/z* 901.74 (**B**), *m/z* 761.92 (**D**), *m/z* 1125.71 (**F**), *m/z* 963.48 (**H**)

Intact glycolipids were further analyzed by MALDI-TOF MS (Fig. 3; Table 3).

**Table 3:** The MALDI-TOF MS spectra of the *C. acnes* glycolipids (GL1-GL4)

	Measured molecular mass (*m/z)*	Ion interpretation	Calculated monoisotopic mass	Components	Chemical formula
GL1	979.55	[M+(CH_2_)_4_+2Na-H]^+^	934.66	2Glc + Gro+17:0 + 18:0	C_50_H_94_O_15_
	951.77	[M+(CH_2_)_2_+2Na-H]^+^	906.63	2Glc + Gro+16:0 + 17:0	C_48_H_90_O_15_
	935.72	[M+(CH_2_)_4_+H]^+^	934.66	2Glc + Gro+17:0 + 18:0	C_50_H_94_O_15_
	923.73	[M+2Na-H]^+^	878.59	2Glc + Gro+15:0 + 16:0	C_46_H_86_O_15_
	901.74	[M + Na]^+^	878.59	2Glc + Gro+15:0 + 16:0	C_46_H_86_O_15_
	891.67	[M-CH_2_-H_2_O+2Na-H]^+^	864.58	2Glc + Gro+15:0 + 15:0	C_45_H_84_O_15_
	883.61	[M-H_2_O + Na]^+^	878.59	2Glc + Gro+15:0 + 16:0	C_46_H_86_O_15_
	847.64	[M-CH_2_-H_2_O + H]^+^	864.58	2Glc + Gro+15:0 + 15:0	C_45_H_84_O_15_
	833.64	[M-(CH_2_)_2_-H_2_O + H]^+^	850.57	2Glc + Gro+15:0 + 14:0	C_44_H_82_O_15_
GL2	831.84	[M+(CH_2_)_5_+2Na-H]^+^	786.62	Glc + Gro+18:0 + 18:0	C_45_H_86_O_10_
	817.83	[M+(CH_2_)_4_+2Na-H]^+^	772.61	Glc + Gro+17:0 + 18:0	C_44_H_84_O_10_
	803.69	[M+(CH_2_)_3_+2Na-H]^+^	758.59	Glc + Gro+16:0 + 18:0	C_43_H_82_O_10_
	795.62	[M+(CH_2_)_4_+Na]^+^	772.61	Glc + Gro+17:0 + 18:0	C_44_H_84_O_10_
	789.76	[M+(CH_2_)_2_+2Na-H]^+^	744.57	Glc + Gro+16:0 + 17:0	C_42_H_80_O_10_
	781.63	[M+(CH_2_)_3_+Na]^+^	758.59	Glc + Gro+16:0 + 18:0	C_43_H_82_O_10_
	767.78	[M+(CH_2_)_2_+Na]^+^	744.57	Glc + Gro+16:0 + 17:0	C_42_H_80_O_10_
	761.92	[M+2Na-H]^+^	716.54	Glc + Gro+15:0 + 16:0	C_40_H_76_O_10_
	745.66	[M+(CH_2_)_2_+H]^+^	744.57	Glc + Gro+16:0 + 17:0	C_42_H_80_O_10_
	739.73	[M + Na]^+^	716.54	Glc + Gro+15:0 + 16:0	C_40_H_76_O_10_
GL3	1175.72	[M+(CH_2_)_2_+2Na-H]^+^	1130.84	2Glc + Gro+16:0 + 16:0 + 16:0	C_63_H_118_O_16_
	1147.69	[M+2Na-H]^+^	1102.81	2Glc + Gro+15:0 + 16:0 + 15:0	C_61_H_114_O_16_
	1125.71	[M + Na]^+^	1102.81	2Glc + Gro+15:0 + 16:0 + 15:0	C_61_H_114_O_16_
	1079.67	[M-(CH_2_)_2_-H_2_O + Na]^+^	1074.78	2Glc + Gro+14:0 + 15:0 + 15:0	C_59_H_110_O_16_
GL4	1042.05	[M+(CH_2_)_4_+2Na-H]^+^	996.82	Glc + Gro+18:0 + 16:0 + 16:0	C_59_H_112_O_11_
	1014.01	[M+(CH_2_)_2_+2Na-H]^+^	968.79	Glc + Gro+16:0 + 16:0 + 16:0	C_57_H_108_O_11_
	999.99	[M+CH_2_+2Na-H]^+^	954.77	Glc + Gro+16:0 + 15:0 + 16:0	C_56_H_106_O_11_
	992.02	[M+(CH_2_)_2_+Na]^+^	968.79	Glc + Gro+16:0 + 16:0 + 16:0	C_57_H_108_O_11_
	985.97	[M+2Na-H]^+^	940.76	Glc + Gro+16:0 + 15:0 + 15:0	C_55_H_104_O_11_
	963.48	[M + Na]^+^	940.76	Glc + Gro+16:0 + 15:0 + 15:0	C_55_H_104_O_11_
	949.75	[M-CH_2_+Na]^+^	926.74	Glc + Gro+15:0 + 15:0 + 15:0	C_54_H_102_O_11_
	935.77	[M-(CH_2_)_2_+Na]^+^	912.73	Glc + Gro+15:0 + 14:0 + 15:0	C_53_H_100_O_11_
	927.70	[M-CH_2_+H]^+^	926.74	Glc + Gro+15:0 + 15:0 + 15:0	C_54_H_102_O_11_

GL1. The major fraction represented by the ions at *m/z* 923.73 [M+2Na-H]^+^, *m/z* 901.74 [M + Na]^+^and *m/z* 883.61 [M-H_2_O + Na]^+^ corresponded to the structure containing two glucose residues (Glc); glycerol (Gro) and two fatty acid residues: 15:0 and 16:0. Also the ions represented structures related to the presence of different fatty acid residues have been identified (Fig. 3A). The ion at *m/z* 901.74 was selected for further analysis by use of positive ion mode MALDI-TOF MS/MS. The main daughter ions detected in the MALDI-TOF MS/MS spectrum were identified (Fig. 3B). The ion at *m/z* 277.1 [M + Na]^+^ corresponds to the GlcGro fragment, while the ion at *m/z* 497.5 to GlcGro16:0 [M-H_2_O + Na]^+^, and the ion at *m/z* 659.6 to GlcGlcGro16:0 [M-H_2_O + Na]^+^ fragments. The ion at *m/z* 739.8 [M-H_2_O + Na]^+^ was assigned to GlcGro16:0_15:0. These observations, in comparison with NMR data and fatty acid analysis, indicate that these fatty acid residues substitute to Gro.

GL2. The major fraction represented by the ions at *m/z* 761.92 [M+2Na-H]^+^, and *m/z* 739.73 [M + Na]^+^ corresponded to the structure containing glucose residue (Glc); glycerol (Gro) and two fatty acid residues: 15:0 and 16:0. Other ions correspond to structures with mass differences related to fatty acids heterogeneity (Fig. 3C; Table 3). The ion at *m/z* 761.92 was selected for further analysis by MALDI-TOF MS/MS (Fig. 3D). The major ion at *m/z* 519.5 is attributed to the loss of pentadecanoic acid, GlcGro16:0 [M-H_2_O+2Na-H]^+^. The ions at *m/z* 341.3 and 385.4 correspond to Gro18:0 fragment [M-H_2_O + H]^+^and [M-H_2_O+2Na-H]^+^, respectively. The comparison with the other presented data indicates the Gro substitution by two fatty acid residues in the GL2.

GL3. The major fraction represented by the ions at *m/z* 1147.69 [M+2Na-H]^+^, and at *m/z* 1125.71 [M + Na]^+^ corresponded to the structure containing two glucose residues (Glc); glycerol (Gro) and three fatty acid residues: two 15:0 and 16:0 (with a total chain length of 46 carbons) (Fig. 3E; Table 3). NMR data have shown the substitution in this structure on 3,4)-β-D-Glc*p* (residue D) and two on the Gro residue. The ion at *m/z* 1125.71 was selected for further analysis (Fig. 3F). The ions at *m/z* 721.6 and at *m/z* 883.6 correspond to GlcGro15:0_16:0 [M-H_2_O + Na]^+^ and GlcGlcGro15:0_16:0 fragments [M-H_2_O + Na]^+^, respectively. The ion at *m/z* 963.8 corresponds to the GlcGro15:0_15:0_16:0. The MALDI-TOF MS analysis in comparison with NMR data indicates Gro substitution by two fatty acid residues (Table 3).

GL4. The major fraction represented by the ions at *m/z* 985.97 [M+2Na-H]^+^, at *m/z* 963.48 [M + Na]^+^ corresponded to the structure containing glucose residue (Glc); glycerol (Gro) and three fatty acid residues with a total chain length of 46 carbons, most likely consisting of two pentadecanoic acids (15:0) and hexadecanoic acid (16:0) (Fig. 3G; Table 3). Additionally, ions at *m/z* 1014.01, and at *m/z* 1042.05, correspond to the structures substituted by different lengths of fatty acids. The ion at *m/z* 963.48 was selected for further analysis (Fig. 3H). The ions at *m/z* 721.8 and at *m/z* 563.8 correspond to GlcGro15:0_16:0 and Gro15:0_15:0 fragments, respectively. Moreover, the ion at *m/z* 441.5 corresponds to Glc16:0 [M + Na]^+^. The MALDI-TOF MS analysis in comparison with NMR data indicates β-D-Glc*p* substituted by a fatty acid residue at the 4 position and Gro substitution by two fatty acid residues, respectively.

To confirm the results obtained by MALDI-TOF MS/MS, we analyzed the glycolipids GL1-GL4 using ESI-IT-MS. The MS and MS/MS spectra for glycolipids GL1, GL2, and GL4 were recorded in negative ion mode, which proved to be easier to interpret (see Table S1 and Fig. S1). In the negative ESI spectrum, the major ions were identified as [M-H]⁻ ions. For GL1, the ion observed at *m/z* 877.62 corresponds to a structure composed of 2 glucoses, glycerol, and fatty acids with chain lengths of 15:0 and 16:0 (C46H86O15). For GL2, the ion at *m/z* 715.56 is associated with a structure containing glucose, glycerol, and the same fatty acids (C40H76O10). Lastly, for GL4, the ion at *m/z* 939.78 represents a structure with glucose, glycerol, and two 15:0 fatty acids alongside one 16:0 fatty acid (C55H104O11) (see Fig. S1). The proposed fragment ion structures in the ESI MS2 spectra are detailed in Table S1. Unfortunately, we could not obtain reliable MS spectra for glycolipid GL3 in either negative or positive ion modes.

### *C. acnes* EVs lipid profiles

**Fig. 4:**
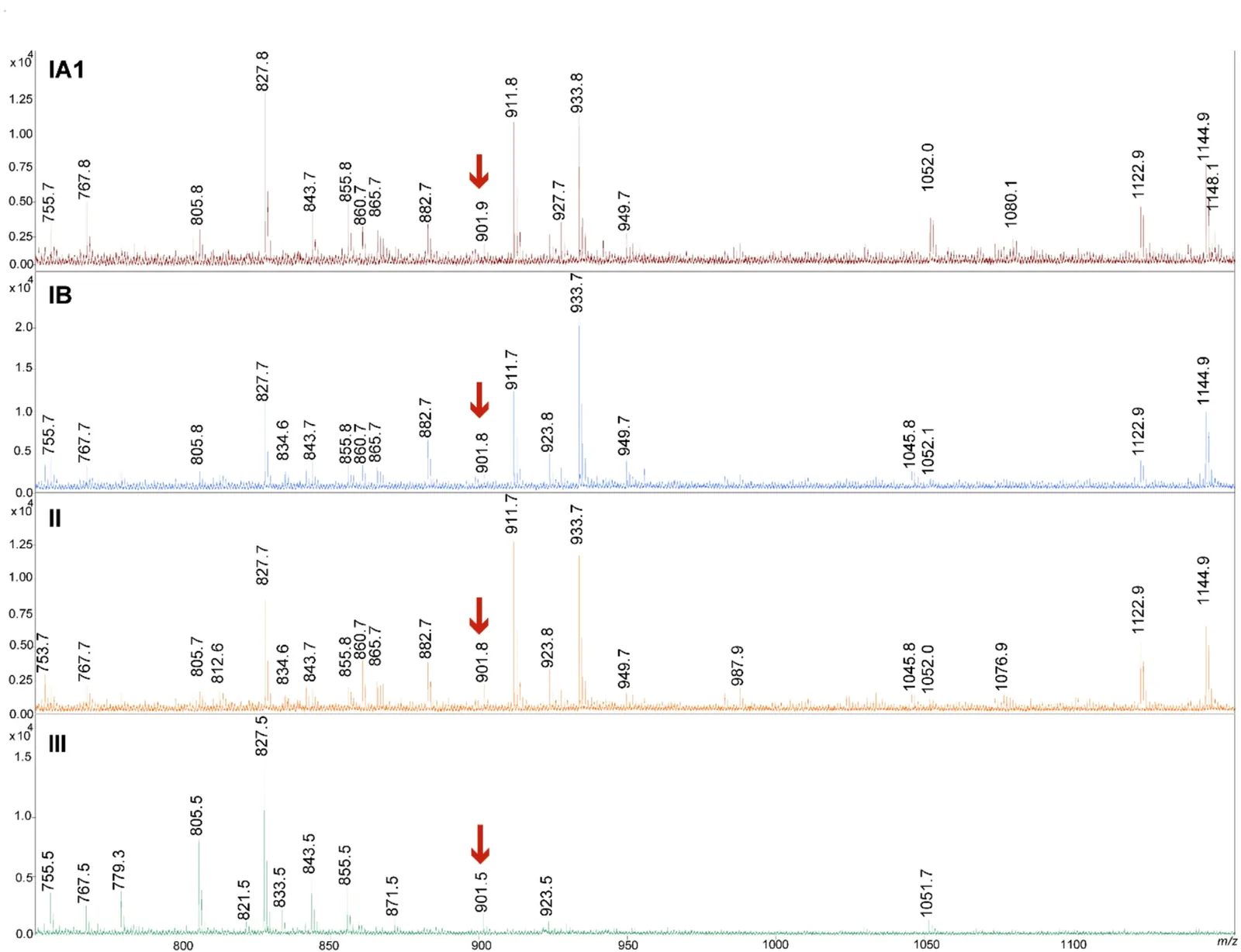
MALDI-TOF MS spectra of EVs lipid extracts isolated from *C. acnes* phylotypes: IA1, IB, II, and III. Mass peaks corresponding to glycolipid GL1 are marked in red

In our previous investigation, EVs from *C. acnes* phylotypes IA1, IB, and II [14] have been isolated and characterized. In this study, we focused additionally on EVs from *C. acnes* phylotype III. Four EV lipid extracts were analyzed by MALDI-TOF MS. The ions at *m/z* 901.9, at *m/z* 901.8, at *m/z* 901.8, and at *m/z* 901.5 [M + Na]^+^, corresponded to GL1 (Fig. 4, red arrow) in phylotypes IA1, IB, II and III, respectively.

### Evaluation of the effect of glycolipids and EVs on human cells

Considering the application of glycolipids and EVs as vaccine components, the most important parameter is their safety for eukaryotic cells. We chose fibroblasts and prostate tissue as healthy skin and inflammatory tissue components, respectively.

#### Cell viability

Both major glycolipids GL1 and GL3, and purified EVs obtained from all four *C. acnes* phylotypes, were used to stimulate human fibroblast MSU-1.1 and prostate epithelial PWR-1E cells. First, the toxicity of the main glycolipids GL1 and GL3 towards human fibroblasts and prostate epithelium was verified at concentrations of 0.0002 µg/ml to 20 µg/ml.

**Fig. 5:**
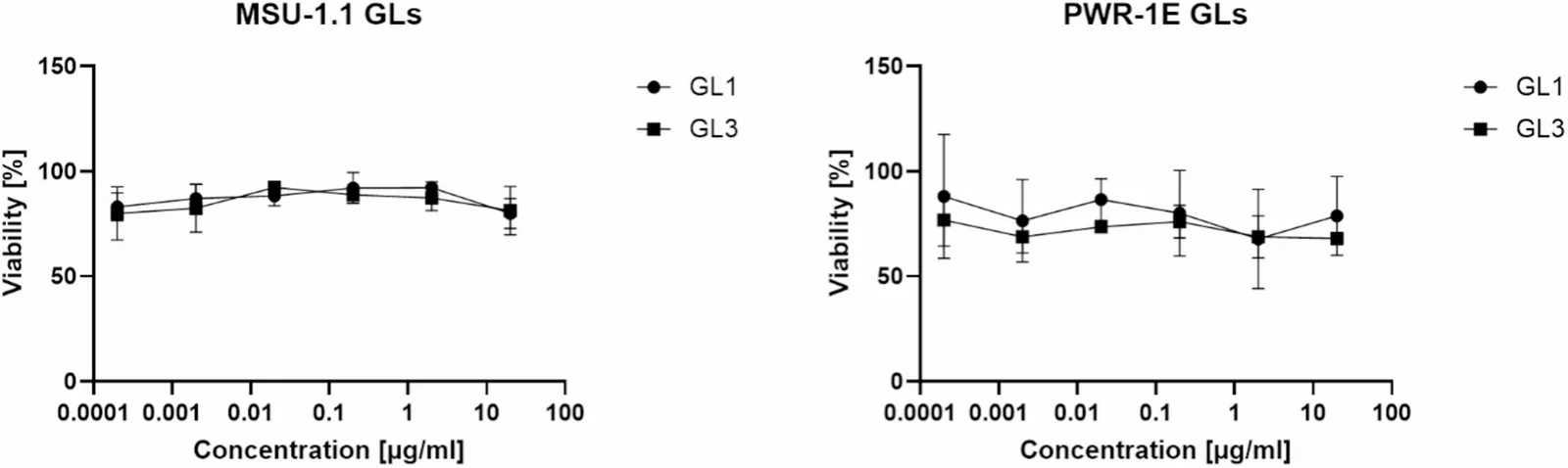
Cytotoxicity of GL1 and GL3 against human cell lines MSU-1.1 and PWR-1E. Unstimulated cells were used as controls (100% viability)

No toxicity of the tested glycolipids was observed on MSU-1.1 fibroblasts, with low decreases in viability ranging from 17.28 to 20.81% in the tested concentration range (Fig. 5). In the case of the PWR-1E line, similar low viability drops of 9.05 to 30.14% were noted.

**Fig. 6:**
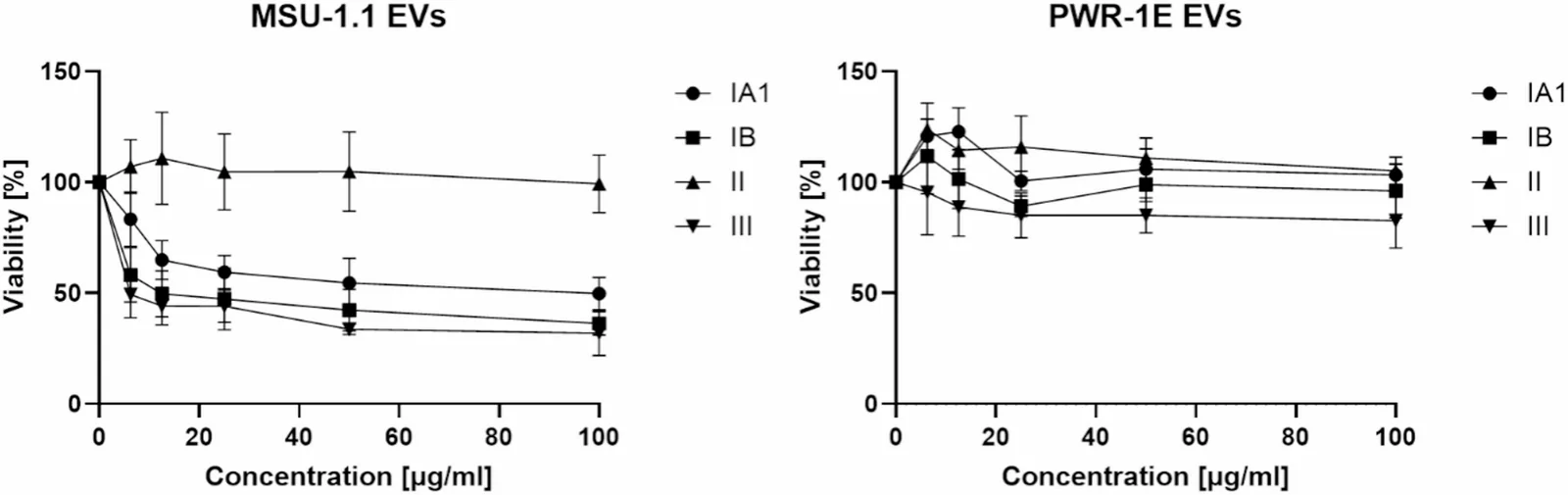
Cytotoxicity of EVs isolated from *C. acnes* phylotype IA1, IB, II and III against human cell lines MSU-1.1 and PWR-1E. Unstimulated cells were used as controls (100% viability)

In the next step, the toxicity of EVs was verified at concentrations of 6.25 µg/ml to 100 µg/ml (Fig. 6).

A statistically significant decrease in the viability of MSU-1.1 fibroblasts stimulated with EVs from phylotypes IA1, IB, and III was observed even at the lowest tested concentration of 6.25 µg/mL (*p* = 0.0325, *p* < 0.0001, and *p* < 0.0001, respectively). A statistically significant decrease in viability was also observed at all tested concentrations for these phylotypes (*p* < 0.0001). The exceptions are EVs isolated from phylotype II, which are non-toxic to these cells (109.28% viability at 6.25 µg/ml). A completely different response to EVs stimulation was observed in prostate epithelial cells PWR-1E, where there was no reduction in viability over the entire range of EVs concentrations.

#### Levels of pro-inflammatory cytokines

The immunomodulatory properties of both *C. acnes* EVs and the major glycolipids GL1 and GL3 were verified using supernatants collected after stimulation with these agents in the concentration range used to determine viability. For this purpose, the levels of cytokines IL-1β, IL-6, and TNF-α were determined using a multiplex assay. Both human cell lines tested, MSU-1.1 fibroblasts and PWR-1E prostate epithelium, did not produce IL-1β in response to stimulation with EVs or GLs.

**Fig. 7:**
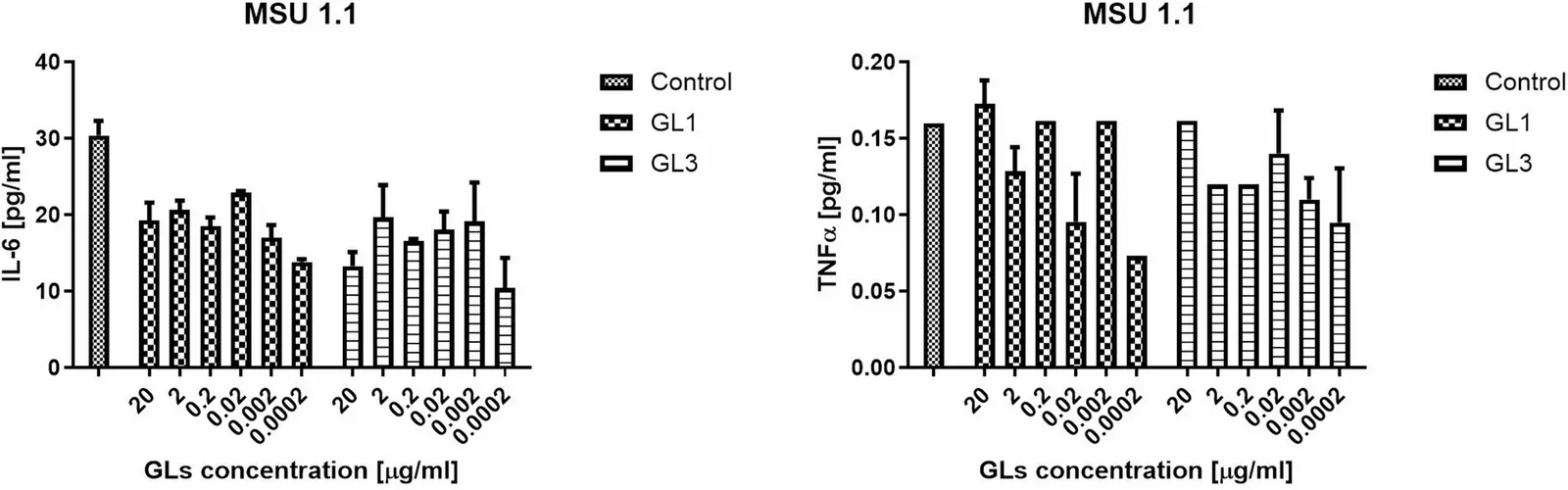
MSU-1.1 fibroblasts IL-6 and TNF-α levels in response to stimulation with *C. acnes* GL1 and GL3 at concentrations of 0.0002–20 µg/ml relative to unstimulated cells (control). ns *p* > 0.05, *p < 0.05, **p < 0.01, ***p < 0.001 vs. non-stimulated controls. The absence of labels/asterisks indicates non-significant differences (*p* > 0.05)

**Fig. 8:**
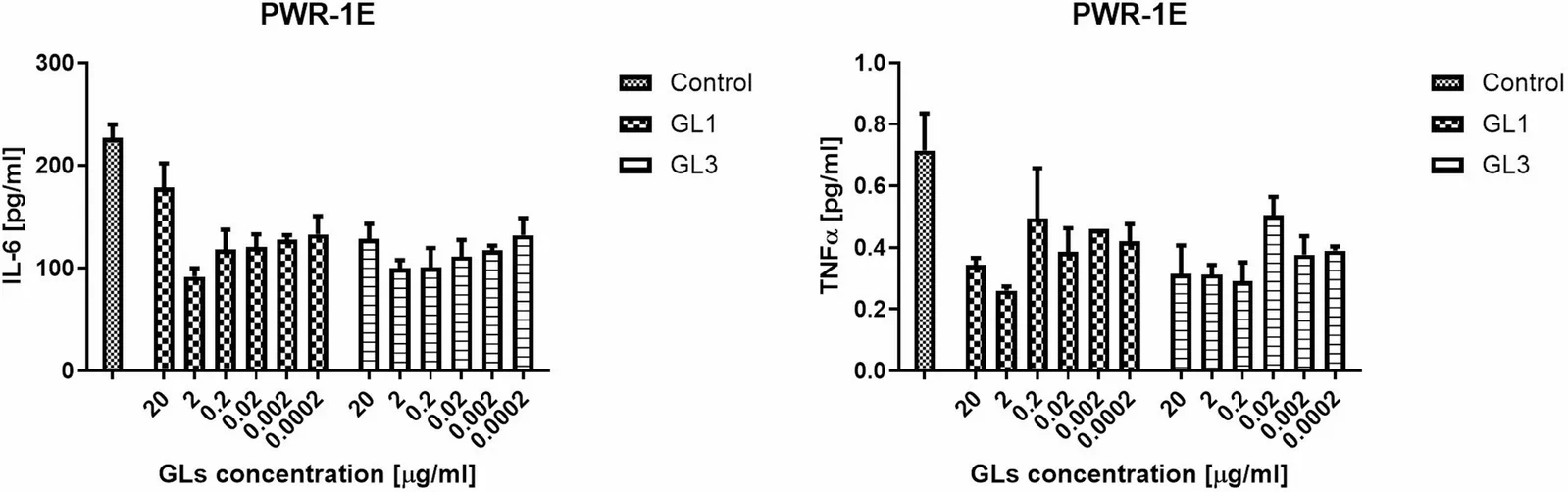
PWR-1E prostate epithelial IL-6 and TNF-α levels in response to stimulation with *C. acnes* GL1 and GL3 at concentrations of 0.0002–20 µg/ml relative to unstimulated cells (control). ns *p* > 0.05, *p < 0.05, **p < 0.01, ***p < 0.001 vs. non-stimulated controls. The absence of labels/asterisks indicates non-significant differences (*p* > 0.05)

Neither of the two glycolipids was shown to induce IL-6 and TNF-α secretion in human fibroblasts or prostate epithelial cells in the entire concentration range studied (0.0002–20 µg/ml). The observed levels of cytokine secretion are similar to those of unstimulated cells (Figs. 7 and 8).

**Fig. 9:**
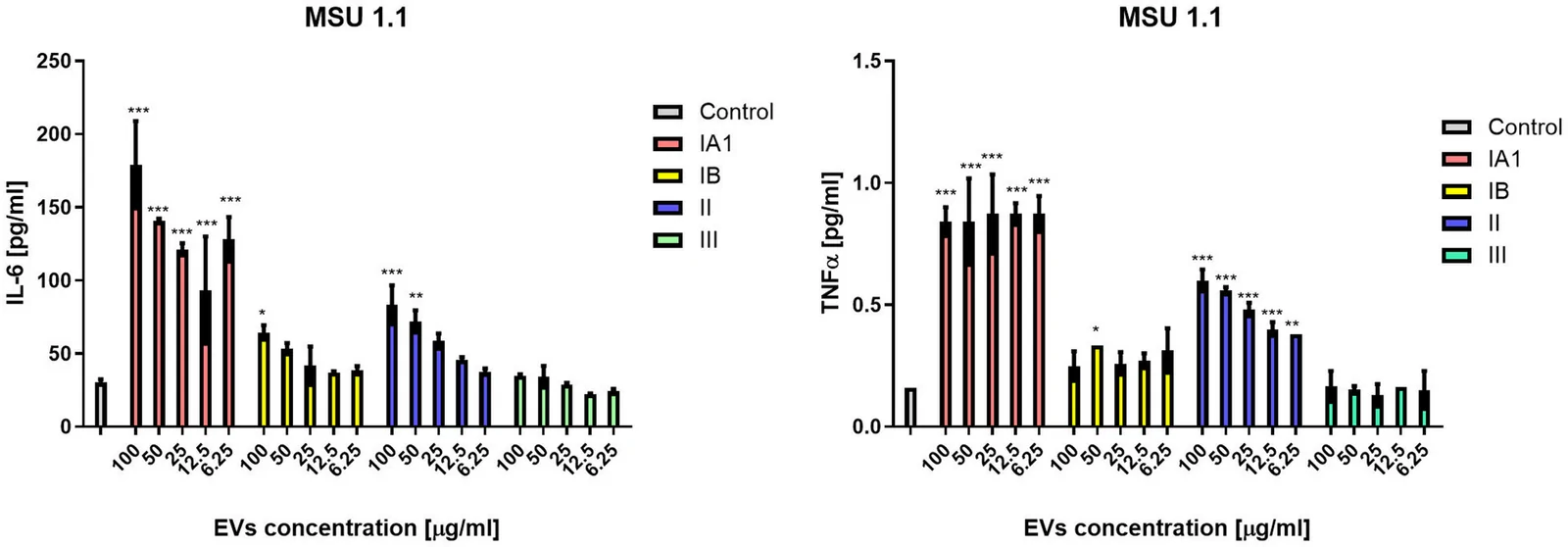
MSU-1.1 fibroblasts IL-6 and TNF-α levels in response to stimulation with *C. acnes* EVs (IA1, IB, II and III) at concentrations of 6.25–100 µg/ml relative to unstimulated cells (control). ns *p* > 0.05, *p < 0.05, **p < 0.01, ***p < 0.001 vs. non-stimulated controls. The absence of labels/asterisks indicates non-significant differences (*p* > 0.05)

**Fig. 10:**
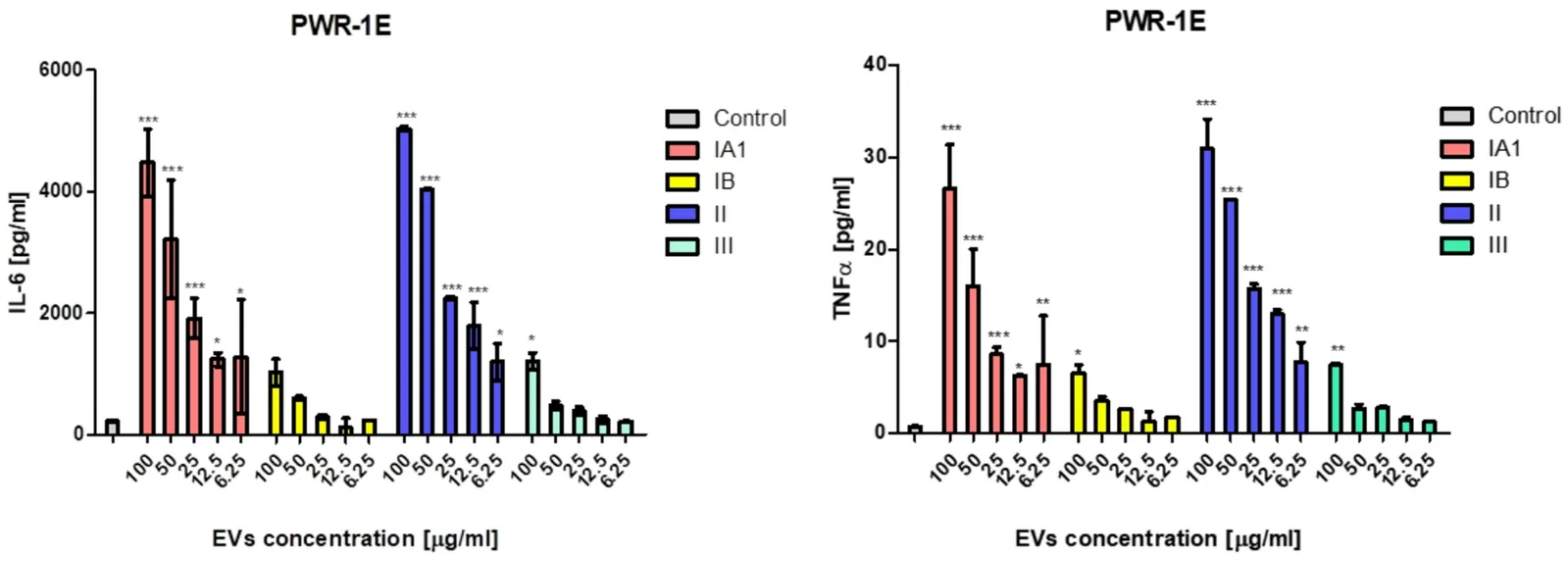
PWR-1E prostate epithelial IL-6 and TNF-α levels in response to stimulation with *C. acnes* EVs (IA1, IB, II and III) at concentrations of 6.25–100 µg/ml relative to unstimulated cells (control). ns *p* > 0.05, *p < 0.05, **p < 0.01, ***p < 0.001 vs. non-stimulated controls. The absence of labels/asterisks indicates non-significant differences (*p* > 0.05)

However, statistically significant differences were observed in the secretion of IL-6 and TNF-α cytokines in response to EVs relative to unstimulated control cells (Figs. 9 and 10).

The highest release of both proinflammatory cytokines released by MSU-1.1 cells was observed after stimulation with EVs from *C. acnes* phylotype IA1 in the entire range of tested concentrations. The second phylotype producing statistically significant proinflammatory EVs was phylotype II. Significantly lower pro-inflammatory potential characterized EVs isolated from *C. acnes* IB and III (Fig. 9).

The results for the PWR-1E cell line confirm the highest pro-inflammatory potential of *C. acnes* EVs IA1 and II, while in the case of this cell line, it is phylotype II that is the strongest dose-dependent stimulant (Fig. 10). The response of prostate epithelial cells to EVs stimulation was several times higher than that of fibroblasts. Again, the low level of secretion of both tested cytokines after stimulation with EVs from phylotypes IB and III, compared to IA1 and II, is visible.

In the case of the MSU-1.1 cell line stimulated with EVs, the highest proinflammatory potential of phylotype IA1 is evident, as indicated by the statistically significantly higher levels of IL-6 and TNF-α compared to the other phylotypes across the entire range of concentrations tested (Fig. S2). This confirms a distinct proinflammatory effect of phylotype IA1 EVs on skin fibroblast cells, which is consistent with reports highlighting the significance of this phylotype in the development of *acne vulgaris*. A distinct situation is observed in the case of the PWR-1E cell line. Here, the most pronounced proinflammatory potential, expressed by the levels of the cytokines IL-6 and TNF-α, is demonstrated by phylotype II EVs, followed by phylotype IA1. The levels of these cytokines obtained after stimulation with phylotype II EVs are statistically significantly higher than those after stimulation with IB and III phylotype EVs. This highlights a distinct involvement of phylotype II EVs in proinflammatory processes within prostate epithelial cells (Fig. S3).

## Discussion

Recent studies on *C. acnes* have highlighted the significance of intensive research into the pathogenicity of these bacteria, not only in the context of *acne vulgaris* but also in persistent prostate inflammation or perioperative infections [29, 30]. One of the key points of interest in microbiology is the direct interactions between bacterial cells, their components, and the particles they secrete into the extracellular environment, which ultimately affect host cells. In *C. acnes*, glycoglycerolipids, particularly diglycosyl acylglycerols, have been identified as key cellular membrane components. This work, for the first time, characterizes the structure of *C. acnes* glycolipids.

These GL1-GL4 glycolipids consist of one or two glucose residues, which are further acylated in GL3 and GL4. They also contain a glycerol molecule that is substituted with two fatty acid residues, each with a chain length of 15 to 18 carbons, with *iso* and *anteiso* methyl branching. All the identified glycolipids are structurally very similar, suggesting that the mechanisms involved in their biosynthesis may be significant for *C. acnes*. Glycolipids GL1 and GL3 were found in all *C. acnes* phylotypes studied. However, the IB phylotype also included additional glycolipids GL2 and GL4. Glycolipids GL2 and GL4 are modified forms of diglucosyl glycerolipids GL1 and GL3, created by cleaving the distal glucose residue. The exact enzymatic cascade responsible for the biosynthesis of these specific glycolipids across the different *C. acnes* phylotypes have not yet been fully elucidated in the literature. While global genomic differences among *C. acnes* phylotypes have been documented [5, 9], the precise metabolic pathways governing glycolipid production remain an open area of research in bacterial lipidomics.

The phylotypes IB and III are mostly expressed in healthy skin. In healthy skin, phylotype IA1 is the most abundant, making up about 40% of isolates, followed by phylotypes II, III, IB, and IA2. In inflammatory acne lesions, IA1 increases to over 70% of *C. acnes* phylotypes, while IB and II decrease significantly. The function of GL1-GL4 glycolipids in individual *C. acnes* phylotypes remains to be elucidated. Exploring the comparative glycolipid biosynthesis and distribution across individual *C. acnes* phylotypes and even the entire *Cutibacterium* genus represents an excellent and highly promising direction for future research.

It is worth mentioning that dihexosyl diacylglycerol (DHDAG) lipids are widespread among Gram-positive bacteria, including genera such as *Staphylococcus*,* Streptococcus*,* Enterococcus*, and *Bacillus* [31–33]. The carbohydrates involved—primarily glucose, galactose, and mannose—can exist in different anomeric configurations, with linkages such as 1–2, 1–3, 1–4, or 1–6. The fatty acids attached to the glycerol moiety can vary in several ways: they may be short or long, simple or branched, and saturated or unsaturated. In *Staphylococcus aureus*, a glycolipid similar to *C. acnes* GL1, known as β-gentobiosyldiacylglycerol, was identified, differing by the 1–6 linkage [32]. Other glycoglycerolipids have been discovered in various species within the *Propionibacteriaceae* family. For instance, *Pseudopropionibacterium propionicum* (formerly known as *Propionibacterium propionicum*) produces glycerolipids in its membranes that have a similar structure but feature ether-linked alkyl chains [34].

In the subsequent stage of the research, the presence of glycerolipids in the extracellular vesicles (EVs) released by *C. acnes* into the culture media was investigated. A MALDI-TOF MS analysis of lipid extracts from EV fractions obtained from four distinct phylotypes of *C. acnes* was conducted, revealing the presence of glycolipid GL1, a constituent of the bacterial membrane, within these extracts. Subsequently, an evaluation was conducted to ascertain the impact of glycolipids and EVs on human cells. The primary focus of this evaluation was on the assessment of cell viability and the induction of pro-inflammatory cytokines.

Bacterial EVs are an excellent natural carrier of lipid particles, allowing for the reflection of their interactions with host cells. Taking into account the role of these bacteria in the pathogenesis of *acne vulgaris* and chronic prostatitis, we verified the effect of EVs from four *C. acnes* phylotypes (IA1, IB, II, and III) on fibroblast cells, which naturally build the human dermis, and prostate epithelial cells, which under physiological conditions, are in direct contact with bacteria during prostatitis. It is essential to verify different phylotypes, as studies have indicated the role of phylotype II of *C. acnes* in the development of prostatitis, potentially contributing to the etiology of prostate cancer [35, 36]. It was shown that *C. acnes* II harbors distinct genomic determinants – including tad-encoded Flp (fimbrial low-molecular mass protein) pili – that enhance epithelial adherence and may facilitate long-term niche persistence [35]. Infection of prostate epithelial cells elicits robust, yet spatially restricted, pro-inflammatory signaling characterized by the induction of cytokines such as IL-6 and IL-8, while systemic inflammatory biomarkers often remain unaltered, indicating a largely compartmentalized host response [37]. Moreover, several type II isolates exhibit immunomodulatory or tolerogenic activities, thereby introducing complexity into the mechanistic link between microbial persistence, immune dysregulation, and oncogenic transformation [38]. Emerging molecular data further suggest that *C. acnes* II infection can impair host DNA repair pathways, including homologous recombination processes, thereby fostering genomic instability conducive to prostate carcinogenesis [39]. In the course of this study, we showed that it was the EVs of *C. acnes* phylotype II that most strongly stimulated the proinflammatory response in the PWR-1E prostate epithelial cell line, increasing IL-6 and TNF-α levels, which is consistent with the reports mentioned above.

The interactions of EVs isolated from *C. acnes* with human keratinocytes, which build the epidermis, are well described and have been the subject of many studies [2, 40, 41]. It was shown that these active nanostructures elicit potent pro-inflammatory responses in human keratinocytes, characterized by the robust induction of IL-6, IL-8, GM-CSF and TNF-α. EVs undergo clathrin-mediated internalization and subsequently activate canonical innate immune signaling pathways, including TLR2-dependent MAPK and NF-κB cascades, thereby promoting epidermal dysregulation and acne-associated inflammatory phenotypes [21]. Proteomic and lipidomic profiling have demonstrated that EV cargo composition varies substantially across *C. acnes* phylotypes, suggesting that strain-specific molecular determinants critically modulate vesicle immunogenicity and downstream host responses [2, 14]. Furthermore, pharmacological interventions such as cannabidiol have been shown to attenuate EV-induced inflammatory signaling via CB2 receptor activation, highlighting the translational potential of targeting EV-mediated host–microbe interactions in inflammatory dermatoses [42]. In our study, we decided to verify for the first time the interactions of these nanostructures with deeper layers of the skin using human fibroblasts. Of all four *C. acnes* phylotypes, phylotype IA1 was characterized by the production of EVs with the highest pro-inflammatory potential in the MSU-1.1 skin fibroblast cell line - this is consistent with numerous studies that indicated the involvement of this phylotype in inflammatory processes in the skin, especially in the context of the etiopathogenesis of *acne vulgaris* [4, 43].

In summary, the pronounced pro-inflammatory effect induced by EVs secreted by *C. acnes* phylotypes IA1 and II is most likely attributable to a higher number of virulence factors present in the cells of these phylotypes compared to the others—*C. acnes* IB and III [2, 8].

A significant finding in this study was the lack of immunomodulatory effects following stimulation with purified GL1 and GL3 glycolipids on MSU-1.1 and PWR-1E cells. Likely, the cells used in this experiment do not recognize bacterial glycolipids in this form; further research in this area could be based on placing the tested glycolipids in liposome membranes, which enables natural exposure while using an immunogenically neutral carrier.

## Conclusion

This study allowed for the precise determination of the structures of *C. acnes* glycolipids but also for the demonstration that these glycolipids are incorporated into EVs membranes by Cutibacteria and secreted into the external environment. The next stages of the study enabled the verification of the interactions between these nanostructures and human cells, shedding new light on the etiopathogenesis of diseases such as prostatitis, which may underlie cancer, as well as interactions with the deep layers of the skin. Importantly, this work demonstrated that EVs isolated from different phylotypes of *C. acnes* interact with human cells in a phylotype-dependent manner. This reemphasizes the role of *C. acnes* phylotype IA1 as an important pro-inflammatory factor in the skin and provides further information on the role of *C. acnes* phylotype II and its metabolites in the context of prostate inflammation. While *acne vulgaris* is the most common skin disease in the world, causing primarily cosmetic defects, prostate cancer is the 4th most common cancer in the world, contributing to 396,000 deaths in 2022 [44]. Therefore, searching for the causes of these diseases and precisely determining the basis of inflammatory processes at the cellular level is necessary to understand the mechanisms underlying these diseases and contribute to the search for innovative therapies or vaccines.

The chemical and biological characteristics of *C. acnes* glycolipids provide a foundation for further research into the antigenic properties of these compounds, particularly in the quest for new vaccine components and diagnostic tools. Additionally, exploring the role of glycolipids in extracellular vesicles (EVs) is a very promising area of research.

## Supplementary Information

Supplementary Material 1.

## Data Availability

The raw mass spectrometry data can be accessed in Zenodo EU Open Research Repository under the accession number https://zenodo.org/records/18876480. All other data supporting the findings of this study are available from the corresponding authors upon request.
