# Supplementary material for: Extracellular vesicles from Cutibacterium acnes phylotypes IA1, IB, II and III, which contain glucoglycerolipids, are not cytotoxic but induce a strong pro-inflammatory response in human prostate epithelial and fibroblast cells
Source: BMC Microbiol. 2026 Aug 6;26:963. doi: 10.1186/s12866-026-05490-x (PMC13737691; doi:10.1186/s12866-026-05490-x)
Supplement: Supplementary file 1 [file 12866_2026_5490_MOESM1_ESM.zip › Supplementary Table S1_31_07_26.docx]

**Table S1.** The proposed fragment ion structures in the ESI-IT-MS2 spectra of [M-H]^-^ ions of GlcGlcGro15:0_16:0 at *m/z* 877.62 (**GL1**), GlcGro15:0_16:0 at *m/z* 715.56 (**GL2**), and Glc16:0Gro15:0_15:0 at *m/z* 939.78 (**GL4**).

| Compound | Measured molecular mass (*m/z*) | Ion interpretation | Calculated monoisotopic mass | Components | Chemical formula |
| --- | --- | --- | --- | --- | --- |
| GL1 | 697.52 | [M-H_2_O-H]^-^ | 716.54 | Glc+Gro+16:0+15:0 | C_40_H_76_O_10_ |
|  | 653.36 | [M-H]^-^ | 654.38 | 2Glc+Gro+16:0 | C_31_H_58_O_14_ |
|  | 473.25 | [M-H_2_O-H]^-^ | 492.33 | Glc+Gro+16:0 | C_25_H_48_O_9_ |
|  | 455.23 | [M-2H_2_O-H]^-^ | 492.33 | Glc+Gro+16:0 | C_25_H_48_O_9_ |
|  | 241.16 | [M-H]^-^ | 242.22 | 15:0 | C_15_H_30_O_2_ |
| GL2 | 491.26 | [M-H]^-^ | 492.33 | Glc+Gro+16:0 | C_25_H_48_O_9_ |
|  | 473.25 | [M-H_2_O-H]^-^ | 492.33 | Glc+Gro+16:0 | C_25_H_48_O_9_ |
|  | 455.28 | [M-2H_2_O-H]^-^ | 492.33 | Glc+Gro+16:0 | C_25_H_48_O_9_ |
|  | 241.15 | [M-H]^-^ | 242.22 | 15:0 | C_15_H_30_O_2_ |
| GL4 | 715.52 | [M-H]^-^ | 716.54 | Glc+Gro+16:0+15:0 | C_40_H_76_O_10_ |
|  | 697.51 | [M-H_2_O-H]^-^ | 716.54 | Glc+Gro+16:0+15:0 | C_40_H_76_O_10_ |
|  | 679.50 | [M-2H_2_O-H]^-^ | 716.54 | Glc+Gro+16:0+15:0 | C_40_H_76_O_10_ |
|  | 491.26 | [M-H]^-^ | 492.33 | Glc+Gro+16:0 | C_25_H_48_O_9_ |
|  | 473.25 | [M-H_2_O-H]^-^ | 492.33 | Glc+Gro+16:0 | C_25_H_48_O_9_ |
|  | 455.23 | [M-2H_2_O-H]^-^ | 492.33 | Glc+Gro+16:0 | C_25_H_48_O_9_ |
|  | 241.16 | [M-H]^-^ | 242.22 | 15:0 | C_15_H_30_O_2_ |
