# Supplementary material for: Extracellular vesicles from Cutibacterium acnes phylotypes IA1, IB, II and III, which contain glucoglycerolipids, are not cytotoxic but induce a strong pro-inflammatory response in human prostate epithelial and fibroblast cells
Source: BMC Microbiol. 2026 Aug 6;26:963. doi: 10.1186/s12866-026-05490-x (PMC13737691; doi:10.1186/s12866-026-05490-x)
Supplement: Supplementary file 1 [file 12866_2026_5490_MOESM1_ESM.zip › Supplementary Figure legend 31_07_26.docx]

**Supplementary material**

**Figure legend**

**Figure S1**. ESI-IT-MS spectra of *C. acnes* glycolipids in the negative ion mode: A) MS1 of GL1, B) the MS2 spectra of the [M-H]^-^ ion of GlcGlcGro15:0_16:0 at *m/z* 877.62, C) MS1 of GL2, D) the MS2 spectra of the [M-H]^-^ ion of GlcGro15:0_16:0 at *m/z* 715.56, E) MS1 of GL4, F) the MS2 spectra of the [M-H]^-^ ion of Glc16:0Gro15:0_15:0 at *m/z* 939.78.

**Figure S2**. IL-6 and TNF-α levels secreted by MSU-1.1 cells stimulated with EVs from 4 *C. acnes* phylotypes. Cells were stimulated with five concentrations of EVs (100, 50, 25, 12.5, and 6.25 µg/ml) from phylotypes IA1, IB, II, and III (n=2). Data are presented as mean ± SD. Statistical analysis was performed using an ordinary two-way ANOVA followed by Tukey's multiple comparisons test. Asterisks indicate significant differences between phylotypes within the same concentration group (**p* < 0.05, ***p* < 0.01, ****p* < 0.001, *****p* < 0.0001). The absence of labels/asterisks indicates non-significant differences (*p* > 0.05).

**Figure S3**. IL-6 and TNF-α levels secreted by PWR-1E cells stimulated with EVs from 4 *C. acnes* phylotypes. Cells were stimulated with five concentrations of EVs (100, 50, 25, 12.5, and 6.25 µg/ml) from phylotypes IA1, IB, II, and III (n=2). Data are presented as mean ± SD. Statistical analysis was performed using an ordinary two-way ANOVA followed by Tukey's multiple comparisons test. Asterisks indicate significant differences between phylotypes within the same concentration group (**p* < 0.05, ***p* < 0.01, ****p* < 0.001, *****p* < 0.0001). The absence of labels/asterisks indicates non-significant differences (*p* > 0.05).
